# Impact of sodium glucose linked cotransporter‐2 inhibition on renal microvascular oxygen tension in a rodent model of diabetes mellitus

**DOI:** 10.14814/phy2.14890

**Published:** 2021-06-29

**Authors:** Gregory M. T. Hare, Yanling Zhang, Kyle Chin, Kerri Thai, Evelyn Jacobs, Melina P. Cazorla‐Bak, Linda Nghiem, David F. Wilson, Sergei A. Vinogradov, Kim A. Connelly, C. David Mazer, Roger G. Evans, Richard E. Gilbert

**Affiliations:** ^1^ Department of Anesthesia St. Michael's Hospital University of Toronto Toronto ON Canada; ^2^ Department of Physiology University of Toronto Toronto ON Canada; ^3^ Keenan Research Centre for Biomedical Science in the Li Ka Shing Knowledge Institute St. Michael's Hospital Toronto ON Canada; ^4^ Department of Biochemistry and Biophysics School of Medicine University of Pennsylvania Philadelphia PA USA; ^5^ Department of Medicine Division of Cardiology St. Michael's Hospital University of Toronto Toronto ON Canada; ^6^ Institute of Medical Science University of Toronto Toronto ON Canada; ^7^ Cardiovascular Disease Program Biomedicine Discovery Institute and Department of Physiology Monash University Melbourne Vic Australia; ^8^ Department of Medicine Division of Endocrinology St. Michael's Hospital University of Toronto Toronto ON Canada

## Abstract

**Background:**

The mechanisms whereby inhibitors of sodium‐glucose linked cotransporter‐2 (SGLT2) exert their nephroprotective effects in patients with diabetes are incompletely understood but have been hypothesized to include improved tissue oxygen tension within the renal cortex. The impact of SGLT2 inhibition is likely complex and region specific within the kidney. We hypothesize that SGLT2 inhibitors have differential effects on renal tissue oxygen delivery and consumption in specific regions of the diabetic kidney, including the superficial cortex, containing SGLT2‐rich components of proximal tubules, versus the deeper cortex and outer medulla, containing predominantly SGLT1 receptors.

**Methods:**

We measured glomerular filtration rate (GFR), microvascular kidney oxygen tension (P_k_O_2_), erythropoietin (EPO) mRNA, and reticulocyte count in diabetic rats (streptozotocin) treated with the SGLT2 inhibitor, dapagliflozin. Utilizing phosphorescence quenching by oxygen and an intravascular oxygen sensitive probe (Oxyphor PdG4); we explored the effects of SGLT2 inhibition on P_k_O_2_ in a region‐specific manner, in vivo, in diabetic and non‐diabetic rats. Superficial renal cortical or deeper cortical and outer medullary P_k_O_2_ were measured utilizing excitations with blue and red light wavelengths, respectively.

**Results:**

In diabetic rats treated with dapagliflozin, measurement within the superficial cortex (blue light) demonstrated no change in P_k_O_2_. By contrast, measurements in the deeper cortex and outer medulla (red light) demonstrated a significant reduction in P_k_O_2_ in dapagliflozin treated diabetic rats (*p* = 0.014). Consistent with these findings, GFR was decreased, hypoxia‐responsive EPO mRNA levels were elevated and reticulocyte counts were increased with SGLT2 inhibition in diabetic rats (*p* < 0.05 for all).

**Conclusions:**

These findings indicate that microvascular kidney oxygen tension is maintained in the superficial cortex but reduced in deeper cortical and outer medullary tissue, possibly due to the regional impact of SGLT‐2 inhibition on tissue metabolism. This reduction in deeper P_k_O_2_ had biological impact as demonstrated by increased renal EPO mRNA levels and circulating reticulocyte count.

## Graphical abstract

Our findings indicate that microvascular kidney oxygen tension (P_k_O_2_) is maintained in the superficial cortex but reduced in deeper cortical and outer medullary tissue, possibly due to the regional impact of sodium‐glucose linked cotransporter‐2 inhibition on tissue metabolism. This reduction in deeper P_k_O_2_ had biological impact as demonstrated by increased renal erythropoietin mRNA levels and circulating reticulocyte count.

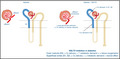

## 1 INTRODUCTION

Several large multicenter randomized controlled trials have shown the ability of sodium‐glucose linked cotransporter‐2 (SGLT2) inhibitors to attenuate the rate of glomerular filtration rate (GFR) decline in patients with diabetes, reduce the incidence of acute kidney injury (Neuen et al., 2019; Perkovic et al., 2019) and improve survival (Inzucchi et al., 2018; Zelniker et al., 2019; Zinman et al., 2015). However, despite these dramatic findings, the mechanisms whereby this new antihyperglycemic drug class exerts its nephroprotective effects are incompletely understood.

Early research by Fine and Norman, bolstered by substantial epidemiological and experimental evidence, suggest that chronic tissue hypoxia has been linked to the development and progression of chronic kidney disease (CKD), including that due to diabetes (Fine et al., 1998; Tanaka et al., 2014). As in other organs, oxygen levels within kidney tissue reflects the combined effects of local oxygen delivery and consumption. Since approximately 80% of the kidney's energy and oxygen requirements are devoted to the reclamation of sodium from the glomerular filtrate, the majority of kidney oxygen consumption occurs in the proximal tubule (Evans et al., 2014; Gilbert, 2017). Changes in sodium reabsorption have a major influence on renal oxygen consumption. Accordingly, improved renal oxygen tension, secondary to reduced oxygen utilization for sodium reabsorption, has been hypothesized as a cogent explanation for the nephroprotective effects of SGLT2 inhibition (Hesp et al., 2020; Kamezaki et al., 2018). However, the consistent finding of increased systemic erythropoietin (EPO) levels and elevated hematocrit after treatment with SGLT2 (Januzzi et al., 2017; McMurray et al., 2019; Zinman et al., 2015), suggest that a decrease in renal PO_2_ may be occurring as a result of SGLT2 inhibition; supporting the rationale for the current study.

The effects of SGLT inhibition on kidney tissue oxygen tension are, notably, complex and multifactorial. In support of this statement, O'Neil et al., have demonstrated that acute treatment with an SGLT1/2 inhibitor (phlorizin) result in an increase in renal sodium excretion and reduced overall oxygen consumption, with differing effects on regional tissue oxygen tension. They demonstrate that phlorizin increased or normalized renal cortical PO_2_ (0.5–1.0 mm probe depth) while simultaneously decreasing medullary tissue PO_2_ (3.5–4.0 mm probe depth) in diabetic rats (O'Neill et al., 2015). These measurements may not have relevance to a nephroprotective mechanism of chronic SGLT2 inhibitors as the therapy was acutely administered, and phlorizin is non‐specific and inhibits both SGLT1 and SGLT2. In addition, the local divergence of oxygen tension in closely approximated regions of the kidney requires re‐evaluation.

**FIGURE 1:**
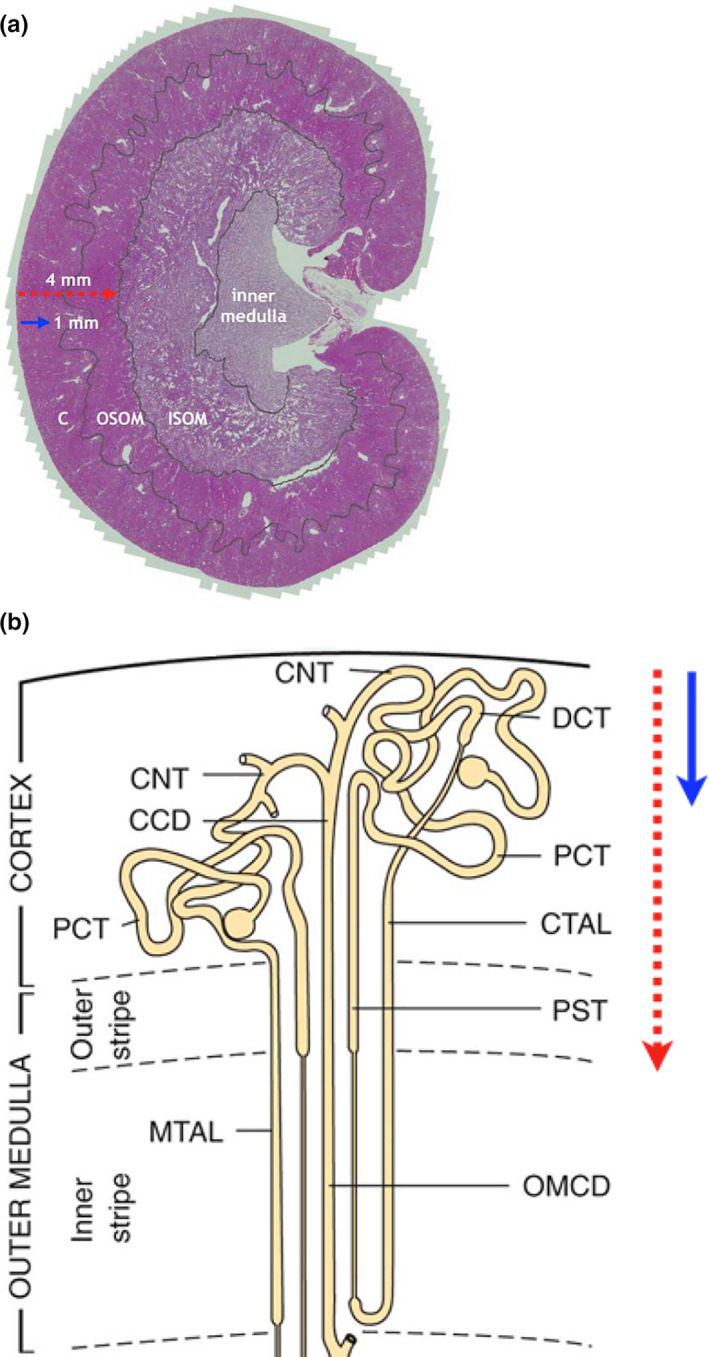
Kidney and nephron anatomical subdivisions. (a) Mid‐sagittal section of rat kidney identifying cortex (C), outer stripe of outer medulla (OSOM), inner stripe of outer medulla (ISOM) and inner medulla. Arrows indicate estimated depth of penetration of red laser light (4 mm) and blue light‐emitting diode (LED) light (1 mm). (b) Diagrammatic illustration of nephron segments within the renal cortex and outer medulla, demonstrating the position of the cortical collecting duct (CCD); connecting tubule (CNT); cortical thick ascending limb (CTAL); distal convoluted tubule (DCT); medullary thick ascending limb (MTAL); outer medullary collecting duct (OMCD); proximal convoluted tubule (PCT); proximal straight tubule (PST). Arrows indicate depth of penetration of red laser and blue LED light (with copyright approval)

With diminished sodium reabsorption in the proximal tubule, specific SGLT2 inhibition would be expected to promote compensatory increases in sodium reabsorption in more distal parts of the nephron. In addition to the energy‐consuming transcellular sodium transport, energy‐independent paracellular transport also contributes substantially to sodium reclamation in the thick ascending loop and to a lesser extent in the proximal tubule but not at all in the distal convoluted tubule, connecting tubule or cortical collecting duct (Mount, 2016). Moreover, while SGLT2 is located in the first two thirds of the proximal tubule (segments S1 and S2), which resides entirely within the cortex, SGLT1 is found in the terminal, pars recta (S3 segment) of the proximal tubule which spans the inner cortex and outer stripe of the outer medulla (OSOM; Figure 1; Chao & Henry, 2010; Kriz & Bankir, 1988). Thus, observations of the physiological impact of dual SGLT1/2 inhibition with agents such as phlorizin (O'Neill et al., 2015) may not be directly transferrable to selective SGLT2 inhibition. Finally, beyond its effects on oxygen consumption, SGLT2 inhibitors would also be predicted to reduce oxygen delivery by increasing afferent arteriolar tone through tubuloglomerular feedback (TGF; Hesp et al., 2020) and potentially decrease efferent arterial flow; thereby reducing postglomerular perfusion to the tubular network (Lytvyn et al., 2017).

In light of these complex and ostensibly opposing effects on kidney oxygenation, in the present study we examined the effects of selective SGLT2 inhibition using a quantitative method of phosphorescence quenching by oxygen for non‐invasive, in vivo, measurement of microvascular oxygen tension in the kidney (P_k_O_2_). We sought to determine regional differences by examining both the superficial cortex (S1 and S2 segment) as well as the area that spans the entire cortex and outer medulla so as to include the deeper, uninhibited, S3 segment of the proximal tubule and mTAL (Figure 1). We hypothesize that SGLT2 inhibitors have differential effects on renal tissue oxygen delivery and consumption in specific regions of the diabetic kidney including the superficial cortex, containing SGLT2‐rich components of proximal tubules versus the deeper outer medulla, containing predominantly SGLT1 receptors.

## 2 METHODS

### 2.1 Animal experiments

Rats were housed in a temperature‐controlled room (22°C) with a 12 h:12 h light–dark cycle and free access to food and water at St Michael's Hospital Animal Research Vivarium (Toronto, ON, Canada). All animal studies were approved by St. Michael's Hospital Animal Ethics Committee in accordance with the NIH Guide for the Care and Use of Laboratory Animals, Eighth edition (2011).

Eighty‐five, 8‐week‐old male Sprague Dawley rats (Charles River, Montreal, QC) were assigned to receive either 65 mg/kg of streptozotocin (STZ; Sigma) diluted in 0.1 M citrate buffer, or citrate buffer alone (non‐diabetic control) by intraperitoneal injection after an overnight fast. Forty‐eight hours later diabetes was confirmed in animals by a blood glucose concentration >15 mmol/L. Six weeks later (12 weeks post‐STZ or buffer) diabetic and non‐diabetic rats were further randomized to receive either dapagliflozin 0.5 mg/kg (gift of Astra Zeneca) twice a day by oral gavage or vehicle (5% 1‐methyl‐2‐pyrrolidinone, 20% polyethylene glycol at 20 mmol/L) for a 6‐week treatment course.

Of the 85 rats, 32 were assigned to Study 1 in which red light (described below) was used to assess kidney tissue oxygenation in control and diabetic groups receiving either vehicle or dapagliflozin (*n* = 6, 6, 8, 12; control + vehicle, control + dapagliflozin, diabetes + vehicle, diabetes + dapagliflozin, respectively). Twenty‐one rats were assigned to Study 2 in which both blue and red light were used to explore region‐specific oxygenation in diabetic rats receiving either vehicle (*n* = 11) or dapagliflozin (*n* = 12). For both protocols, a step up increase in fraction of inspired oxygen (F_I_O_2_) from 21% to 50% and back to 21% was performed to assess changes in renal microvascular oxygen levels at different arterial oxygen tensions (P_a_O_2_). Arterial blood gas and oximetry data was collected at 21% and 50% F_I_O_2_. Thirty‐two rats were assigned to Study 3 in which kidney mRNA expression and reticulocyte count were examined (*n* = 6, 6, 8, 12 for control + vehicle, control + dapagliflozin, diabetes + vehicle, diabetes + dapagliflozin, respectively).

Hemoglobin A_1c_ was assayed by point‐of‐care technology (A1CNow; PTS Diagnostics). At termination, kidneys were immediately excised, decapsulated, flash frozen in liquid N_2_ and stored at −80°C for later analyses.

### 2.2 Surgical preparation for kidney P_k_O_2_ measurements

In spontaneously breathing rats, anesthesia was induced within an inhalation chamber with 4% isoflurane. Anesthesia was then maintained via tracheostomy with 1.5%–2.0% isoflurane, initially in 21% O_2_ at a total flow rate of 2 L/min. Cannulation of the tail artery was performed for drawing of arterial blood. Heart rate (HR) was measured with electrocardiogram electrodes and the rectal temperature was monitored. A thermoregulatory heating pad was used to maintain rectal temperature near 37°C. A computerized data‐acquisition system (PowerLab ADInstruments Inc.) was used to continually monitor mean arterial pressure (MAP), HR and rectal temperature.

### 2.3 Normoxia‐hyperoxia protocol

Rats underwent spontaneous ventilation at different levels of fractional inspired oxygen (F_I_O_2_) including 21% (baseline), 30% and 50% for 20 min after returning to 21%, to demonstrate responsiveness of renal microvascular pO_2_ (P_k_O_2_) to differing levels of arterial oxygen tension (P_a_O_2_). At each level of inspired oxygen, measurement of P_k_O_2_ was performed by light‐emitting diode (LED) or laser, as detailed below. Arterial blood gases were measured at the end of each period prior to changing the F_I_O_2_. At the end of this experiment, the F_I_O_2_ was returned to baseline (21%) and animals allowed to re‐equilibrate for 30 min, prior to termination by lethal injection (T‐61 overdose) under anesthesia (4% isoflurane).

### 2.4 Microvascular kidney oxygen tension measurements

Microvascular kidney P_k_O_2_ was measured in the left kidney using the phosphorescence quenching method taking advantage of wavelength‐dependent tissue penetration, as previously reported (Chin et al., 2021). In brief, the intravascular oxygen probe Oxyphor PdG4 (Esipova et al. 2011) was deployed in combination with a time‐domain OxyLED phosphorometer (Oxygen Enterprises Ltd.). An oximeter (OxyLED) capable of generating light at 450 nm (blue) and 635 nm (red) was used to excite the Soret and the Q‐bands of the tetrabenzoporphyrin at the core of the probe, respectively. A common light detector was utilized to detect emitted phosphorescence following probe excitation with either blue or red light. This methodology, initially developed by (Rumsey et al. (1997) and adapted by Johannes et al. (2007) takes advantage of the excitation of phosphorescence of PdG2 (spectroscopically identical to PdG4) using blue and red light in order to generate P_k_O_2_ measurements in two regions of a rat kidney: (1) the outer cortex (blue light); and (2) a broader area that spans the entire cortex and outer medulla (red light). After anesthetization the PdG4 probe was injected intravenously. The left kidney was exposed via a dorsal flank incision and the subjacent soft tissue and peri‐renal fat were retracted. The kidney was kept within the retroperitoneal space and in the body cavity to maintain the renal temperature comparable to the core temperature. The animal was kept in a hooded experimental chamber to prevent ambient light from interfering with the measurements.

### 2.5 Light excitation and detection

Excitation light was delivered by two different light sources: a blue (*λ*_max_ = 450 nm) LED with penetration of <1 mm and a red laser (*λ*_max_ = 635 nm) with tissue penetration of up to 4 mm (Ash et al., 2017). Accordingly, red laser penetration would be expected to include the entire cortex and outer stripe of the medulla of an adult rat kidney while the blue LED would be confined to the superficial cortex (Figure 1). For detection, the emission detecting light guide was positioned 1–2 mm directly above the focus of excitation and received input from the tissue at a wavelength of 813 nm. Typically, 200 data collection cycles were averaged in a single measurement (2 ms), and the measurements were performed at 1s intervals. Experimental conditions were deemed acceptable with a signal to noise ratio of phosphorescence decay >2.

### 2.6 Gene expression

Samples of renal tissue were taken from right kidney. The kidney was extracted, decapsulated and divided into the deeper medulla, the cortical and outer medullary area and a section of the whole kidney. The samples were immediately flash frozen in liquid nitrogen and stored at −80°C until analysis. RNA isolation and quantitative polymerase chain reaction (qPCR) were performed by an investigator blinded from the treatment groups. mRNA levels of EPO, glucose transporter 1 (GLUT1) and vascular endothelial growth factor (VEGF). Total RNA was isolated from kidney tissues using TRIzol reagent (Invitrogen) according to the manufacturer's protocol, and RNA was quantified by NanoDrop ND‐2000 Spectrophotometer (Thermo Scientific). Two micrograms of total RNA were used for reverse transcription to cDNA using a High Capacity cDNA Reverse Transcription Kit (ABI Applied Biosystems) following manufacturer's protocol. cDNA was amplified by qPCR (QuantStudio 7 Flex; Applied Biosystems) using PowerUp SYBR Green Master Mix (ABI Applied Biosystems). A list of primers used for qPCR can be found in Table 1, with RPL13a used as the housekeeping gene.

**TABLE 1:** Primers for quantitative PCR

Primers	Sequence
Rat RPL13a F	5′‐GATGAACACGCAACCCGTCTC‐3′
Rat RPL13a R	5′‐CACCATCCGCTTTTTCTTGT‐3′
Rat Glut 1 F	5′‐CACGATACTCAGATAGGACATCC‐3′
Rat Glut 1 R	5′‐ACTGTGGTGTCGCTGTTC‐3′
Rat EPO F	5′‐GCCTGTTCTTCCACCTTCA‐3′
Rat EPO R	5′‐GGAGGCAGAAAATGTCACAATG‐3′
Rat VEGF F	5′‐CCCTGGCTTTACTGCTGTACCT‐3′
Rat VEGF R	5′‐TCCATGAACTTCACCACTTGATG‐3′

### 2.7 Glomerular filtration rate

Rats underwent GFR measurement using a modified fluorescein isothiocyanate (FITC)‐inulin plasma clearance assay (Qi et al., 2004). Rats were tail vein injected with 3.74 µl/g body weight of FITC‐inulin. Tail vein blood was sampled at various time points post‐FITC‐inulin injection. The concentration of this agent was then assayed by its fluorescence with a Spectramax M5e microplate reader (Molecular Devices) with 485 nm excitation and 527 nm emission settings. GFR was calculated using the following two phase, exponential decay curve using non‐linear regression statistics as previously described: GFR = *I*/(*A*/*α* + *B*/*β*), where *I* is the amount of FITC‐inulin injected, *A* and *B* are the *y*‐intercept values for the two decay rates, and *α* and *β* are the decay constants for the distribution and elimination phases.

### 2.8 Reticulocyte count

Red blood cell (RBC) and reticulocyte count were assessed by standard flow cytometry methods.

### 2.9 Statistical analysis

Data that did not significantly violate normality according to the Shapiro‐Wilks test (arterial blood gas, complete blood count, and kidney P_k_O_2_) were subjected to one or two‐way ANOVA, when appropriate. Pairwise comparisons were then made using the Holm–Sidak method or Tukey's test. mRNA expression was analyzed using one‐way ANOVA on ranks and Dunn's post‐hoc test. Two‐tailed *p* < 0.05 was considered statistically significant. All analyses were conducted using Sigmaplot software (Systat Software Inc.).

## 3 RESULTS

### 3.1 Animal model of diabetes mellitus

**FIGURE 2:**
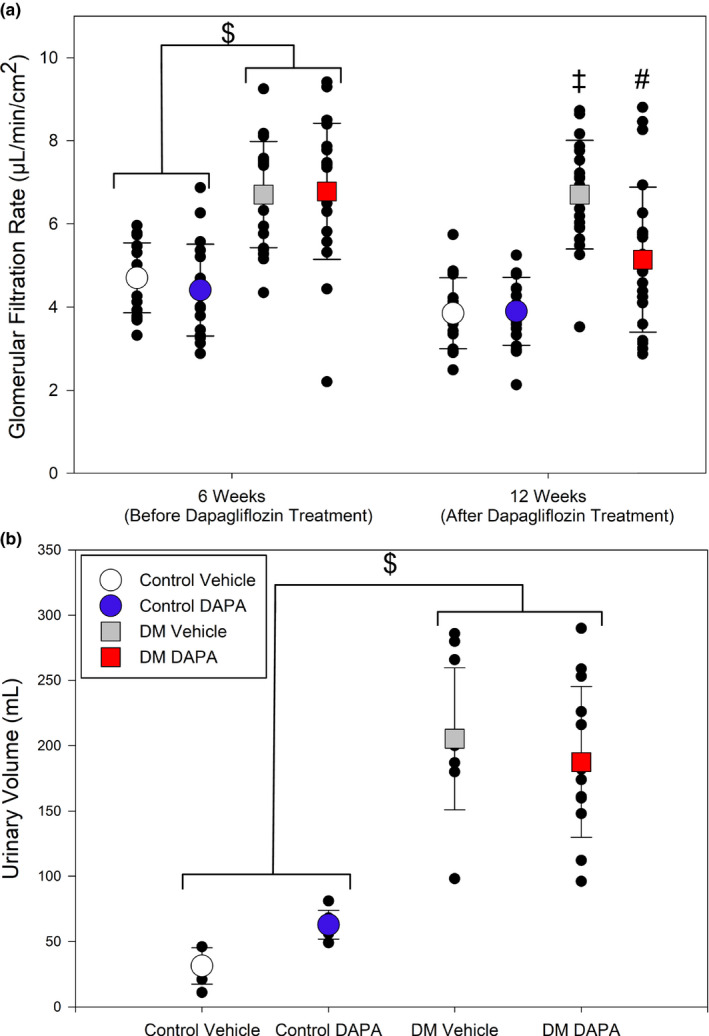
Renal parameters after 6‐week treatment with streptozotocin to induce diabetes, followed by 6 weeks of treatment with dapagliflozin (DAPA), or vehicle. (a) After 6 weeks of streptozotocin treatment, rats developed diabetes mellitus (DM) with an increased glomerular filtration rate (GFR) relative to vehicle‐treated rats ($, *p* < 0.001). At 12 weeks, vehicle‐treated rats with DM maintained significantly higher GFR compared to both non‐diabetic control groups (‡, *p* < 0.004). After 6 weeks of DAPA treatment, the GFR of the DM DAPA group was significantly lower at 12 weeks compared to 6 weeks (#, *p* < 0.001; two‐way repeated measures ANOVA, *n* = 17–22). (b) Diabetic rats produced an increased volume of urine, relative to non‐diabetic rats ($, *p* < 0.02; one‐way ANOVA; *n* = 11–13)

Rats that had received STZ all became diabetic with elevated blood glucose, reduced body weight and elevated hemoglobin A_1c_ > 7% (Table 2). Dapagliflozin treatment reduced blood glucose and HbA_1c_ in diabetic rats, relative to untreated diabetic rats, but did not affect glycemia in non‐diabetic animals (Tables 2 and 3). Dapagliflozin treatment did not change systolic blood pressure for control or diabetic rats. Systolic blood pressure was lower in diabetic animals compared with non‐diabetic dapagliflozin treated rats (*p* = 0.016; Table 2). Diabetic animals showed evidence of hyperfiltration and increased GFR at 6 weeks, prior to treatment with vehicle or dapagliflozin (*p* < 0.001; Table 2; Figure 2, upper panel a). After 6 weeks of administering dapagliflozin (total 12 weeks), GFR was reduced toward baseline in diabetic rats, compared to pretreatment values (*p* < 0.05; Table 2; Figure 2). Rats in both diabetic groups produced more urine than non‐diabetic rats (*p* < 0.02; Figure 2, lower panel b).

**TABLE 2:** Physiological and renal parameters of treatment rats

	Control + vehicle	Control + DAPA	DM + vehicle	DM + DAPA
*N*	6	6	8	12
Body weight (g)	730.8 ± 52.4	713.7 ± 26.4	472.5 ± 32.6*	439.3 ± 20.2*
HbA_1c_ (%)	4.53 ± 0.21	4.65 ± 0.17	12.94 ± 0.04*	9.41 ± 0.62* ^,^ ^†^
Systolic blood pressure (mmHg)	109.7 ± 7.1	124.7 ± 5.1	98.9 ± 5.6^#^	95.9 ± 4.0^#^
GFR (µl/min/g)	4.32 ± 0.34	3.84 ± 0.29	7.54 ± 0.26*	5.84 ± 0.62* ^,^ ^†^
Urine volume (ml/24 h)	33.3 ± 3.6	66.5 ± 2.8	199.4 ± 18.1*	186.9 ± 11.6*
Urinary glucose (mol/24 h)	0.013 ± 0.003	26.31 ± 3.3	91.37 ± 7.8*	78.60 ± 6.6*

Abbreviations: GFR, glomerular filtration rate; HbA_1C_, glycosylated hemoglobin.

* *p* < 0.05 vs. Control + DAPA and Control + vehicle group.

† *p* < 0.05 vs. DM + vehicle group.

# *p* < 0.05 vs. Control + DAPA group (Tukey's test).

**TABLE 3:** Arterial blood gas and cooximetry data from both experimental protocols

F_I_O_2_ (%)	Treatment	pH	pCO_2_ (mmHg)	pO_2_ (mmHg)	HCO_3_ (mmol/L)	Hb (g/L)	sO_2_ (%)	Lac (mmol/L)	Glu (mmol/L)
Experimental protocol #1									
21	Control Vehicle	7.42 ± 0.03	34.6 ± 2.8	80.6 ± 8.6	22.8 ± 0.8	129 ± 7.1	90 ± 2.9	1.63 ± 0.50	12.3 ± 2.5
	Control DAPA	7.42 ± 0.02	36.8 ± 1.3	80.2 ± 8.8	23.8 ± 0.8	130 ± 7.3	89 ± 3.0	1.08 ± 0.25	10.3 ± 2.2^$^
50	Control Vehicle	7.37 ± 0.02*	38.1 ± 2.1	187.0 ± 22*	22.3 ± 1.2	129 ± 8.9	97 ± 0.3	1.77 ± 0.59	10.7 ± 2.9
	Control DAPA	7.37 ± 0.02*	38.1 ± 5.0	202.0 ± 21*	22.1 ± 1.5	125 ± 8.4	97 ± 0.4	0.98 ± 0.37	8.8 ± 1.1^$^
21	Control Vehicle	7.38 ± 0.02	33.0 ± 3.2	73.9 ± 17	20.4 ± 1.0	113 ± 9.0	83 ± 13	2.90 ± 1.94	10.3 ± 4.9
	Control DAPA	7.41 ± 0.02	35.6 ± 3.0	87.9 ± 11	23.4 ± 1.1	128 ± 8.7	91 ± 2.5	1.90 ± 0.44	11.1 ± 1.0^$^
21	DM Vehicle	7.37 ± 0.04^$^, ^ǂ^	34.3 ± 6.6	87.7 ± 21	21.0 ± 3.1	138 ± 8.8	88 ± 2.3	1.52 ± 0.93	21.0 ± 8.2
	DM DAPA	7.30 ± 0.09^$^, ^ǂ^	31.4 ± 5.5	86.5 ± 10	16.4 ± 3.9	122 ± 12	87 ± 3.5	1.69 ± 0.65	12.7 ± 2.9
50	DM Vehicle	7.25 ± 0.10^$^ ^,^ ^ǂ^ ^,^ *	42.1 ± 11	197.7 ± 32*	17.7 ± 4.3^$^, ^ǂ^	136 ± 4.2*	96 ± 0.7*	1.95 ± 1.15*	21.8 ± 2.1^*^
	DM DAPA	7.24 ± 0.09^$^ ^,^ ^ǂ^ ^,^ *	34.0 ± 5.7	213.2 ± 34*	15.7 ± 4.4^#^, ^$^, ^ǂ^	127 ± 11^#^ ^,^ *	97 ± 0.6*	1.54 ± 0.64*	13.3 ± 2.4^*^
21	DM Vehicle	7.30 ± 0.08^$^, ^ǂ^	33.7 ± 7.4	84.7 ± 5.3	17.6 ± 3.9	128 ± 10	88 ± 2.3	1.88 ± 0.74	21.0 ± 2.8
	DM DAPA	7.25 ± 0.09^$^, ^ǂ^	34.2 ± 7.2	85.0 ± 23	16.0 ± 4.6	117 ± 15	82 ± 20	2.13 ± 0.82	14.9 ± 3.3
Experimental protocol #2									
21	DM Vehicle	7.46 ± 0.09	38.4 ± 4.2	66.7 ± 5.5	27.9 ± 7.4	154 ± 6.8	86 ± 2.5	2.0 ± 0.5	28.9 ± 3.6
	DM DAPA	7.41 ± 0.04^#^	34.6 ± 5.3^#^	70.8 ± 7.5	22.4 ± 2.7^#^	143 ± 11^#^	87 ± 4.5	1.8 ± 0.7	16.5 ± 3.5^#^
50	DM Vehicle	7.41 ± 0.03	41.2 ± 3.9*	170.9 ± 26.7*	25.9 ± 1.7	155 ± 5.5	97 ± 1.7*	1.8 ± 0.6*	28.9 ± 4.6
	DM DAPA	7.40 ± 0.08^#^	38.3 ± 3.2^#^ ^,^ *	188.9 ± 15.6*	23.9 ± 5.0^#^	147 ± 8.9^#^	97 ± 0.6*	1.5 ± 0.4*	15.7 ± 4.3^#^
21	DM Vehicle	7.45 ± 0.07	37.2 ± 2.7	75.1 ± 8.3	26.7 ± 6.2	156 ± 4.6	89 ± 2.9	2.5 ± 0.4	28.7 ± 1.5
	DM DAPA	7.40 ± 0.05^#^	35.3 ± 3.8^#^	75.6 ± 6.6	22.4 ± 2.4^#^	146 ± 7.6^#^	87 ± 3.0	2.1 ± 0.7	16.0 ± 4.4^#^

# *p* < 0.05 versus DM vehicle.

$ *p* < 0.05 versus Control DAPA.

ǂ *p* < 0.05 versus Control vehicle.

* *p* < 0.05 versus 21%.

### 3.2 SGLT2 inhibition reduces renal cortical and outer medullary kidney microvascular P_k_O_2_ measured with red light

**FIGURE 3:**
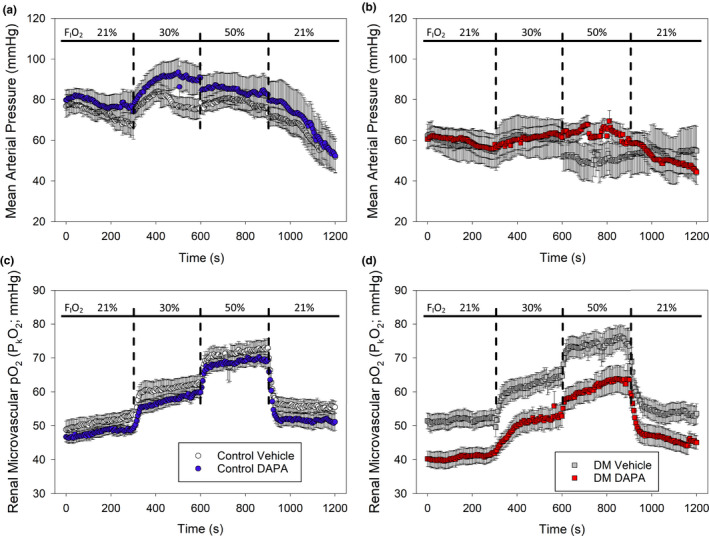
Assessment of the impact of dapagliflozin (DAPA) treatment on real‐time renal microvascular P_k_O_2_ at different levels of inspired oxygen (F_I_O_2_) in non‐diabetic control or diabetic rats (protocol #1). (a) There was no significant difference in mean arterial pressure (MAP) between non‐diabetic control rats treated with vehicle or DAPA. (b) There was no significant difference in MAP between diabetic rats treated with either vehicle or DAPA, at any F_I_O_2_ level. (c) When assessed between groups over time, microvascular tissue oxygen was marginally lower in the control DAPA group relative to control vehicle (*p* = 0.012; two‐way ANOVA). (d) Renal microvascular P_k_O_2_ was significantly lower in diabetic rats treated with DAPA compared to vehicle‐treated diabetic rats (*p* < 0.001; two‐way ANOVA). The microvascular kidney P_k_O_2_ remained about 10 mmHg lower, in DAPA versus vehicle‐treated diabetic rats, at all F_I_O_2_ levels (*n* = 6–9 per group)

**FIGURE 4:**
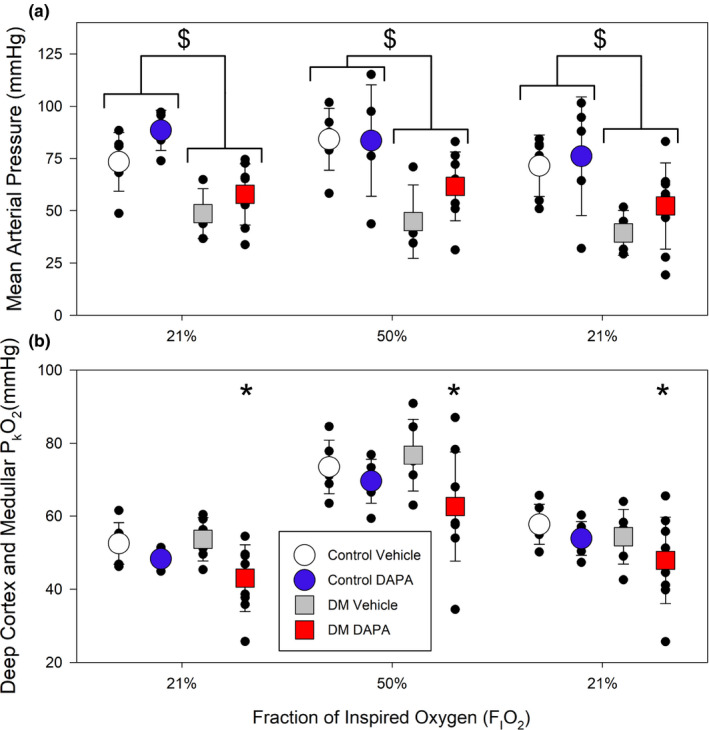
Assessment of the impact of dapagliflozin (DAPA) treatment on real‐time renal microvascular P_k_O_2_ in non‐diabetic control or diabetic rats (protocol #1). (a) When analyzed across all groups, diabetic animals had a lower mean arterial pressure (MAP) relative to control animals ($, *p* < 0.004; two‐way ANOVA with Holm‐Sidak). There was no effect of DAPA treatment relative to vehicle controls. (b) When assessed across all groups, renal microvascular P_k_O_2_ was lower in diabetic rats treated with DAPA, relative to all other groups and at all levels of inspired oxygen (*, *p* = 0.002; two‐way ANOVA). As expected, renal microvascular P_k_O_2_ is higher for all groups at 50% inspired oxygen (*p* < 0.001 vs. 21% F_I_O_2_; two‐way ANOVA; *n* = 6–9 per group)

To determine whether the effects of SGLT2 inhibition differ between the non‐diabetic and diabetic setting, renal microvascular oxygen tension (P_k_O_2_) was determined by examining phosphorescence quenching in response to red light excitation. Using light from a red laser (*λ*_max_ = 635 nm) which penetrates an estimated 4 mm into the kidney (Figure 1; Ash et al., 2017). The red light path is expected to include the cortex and outer medulla. We measured P_k_O_2_ at 21%, 30%, 50% and back to 21% F_I_O_2_ in non‐diabetic and diabetic rats treated with DAPA or vehicle (Figure 3). MAP was not influenced by DAPA treatment (Figures 3 and 4, upper panels). Under anesthesia, MAP was lower for diabetic rats (DAPA and vehicle treated), relative to non‐diabetic rats at all time points (*p* < 0.004, Figure 4, upper panels a, b). In all non‐diabetic and diabetic rats, arterial P_a_O_2_ and renal microvascular P_k_O_2_ changed in proportion to changes in the fraction of inspired oxygen (F_I_O_2_; Figure 3; Table 3). At each level of F_I_O_2_, there was a slight reduction in renal microvascular P_k_O_2_ in non‐diabetic rats treated with DAPA versus vehicle‐treated rats (*p* = 0.012; Figure 3). In diabetic rats, DAPA treatment resulted in a significantly larger reduction in renal microvascular P_k_O_2_ at all F_I_O_2_ levels, relative to vehicle‐treated diabetic rats (*p* < 0.001; Figures 3 and 4, lower panels).

In these experiments, the MAPs were significantly lower in the diabetic rats compared to the control (*p* < 0.004; Figure 4 upper panel), but there was no difference in MAP with DAPA treatment relative to vehicle‐treated non‐diabetic vs diabetic rats (Figures 3 and 4). Arterial pO_2_ values were similar in control and diabetic animals and was not affected by DAPA treatment at any level of inspired oxygen (Table 3).

### 3.3 **SGLT2 inhibition exerts region‐specific effects on kidney oxygenation in diabetic rats**.

**FIGURE 5:**
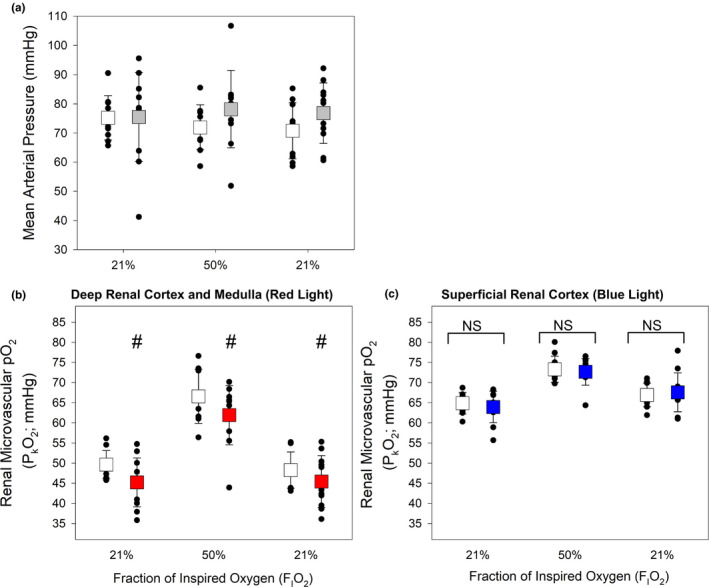
Effect of treatment with dapagliflozin (DAPA) on diabetic rats: assessment of deep (red light) and superficial (blue light) microvascular kidney oxygen tension (P_k_O_2_; protocol #2). (a) No significant differences in mean arterial pressure were observed between DAPA versus Vehicle‐treated diabetic rats. (b) As in protocol #1, diabetic rats treated with DAPA had a significantly lower deep renal cortical and medullary microvascular P_k_O_2_ (red light) relative to vehicle‐treated diabetic rats (#, *p* = 0.018; two‐way ANOVA). (c) By contrast, no differences in the superficial renal cortical P_k_O_2_ (blue light) were observed when comparing diabetic rats treated with DAPA compared to vehicle. (*n* = 10–11 rats per group)

Given the measured reduction in renal cortex and outer medullar oxygen tension (P_k_O_2_) observed in DAPA treated diabetic animals, we next sought to determine whether region‐specific responses were evident in the diabetic setting. To do this, in addition to the red laser we also employed a blue LED light system which, due to its narrower wavelength (*λ*_max_=450 nm), has a reduced tissue penetration of only ~1 mm (Figure 1; Ash et al., 2017). As such, it reflects tissue oxygen tension in the superficial cortex of the rat. In this study, we first confirmed, using the red laser, that DAPA treatment resulted in a reduction in renal P_k_O_2_ within the cortex and outer medulla of diabetic rats (*p* = 0.018; Figure 5, lower panel b). Interrogation with blue light revealed that P_k_O_2_ was notably higher in the superficial cortex than in deeper parts of the kidney assessed with red light. In addition, unlike the reduction in renal P_k_O_2_ observed with red light, no decrease was observed when P_k_O_2_ was measured simultaneously with blue light (*p* = 0.735; Figure 5, lower panel c). This demonstrated a region‐specific reduction in renal P_k_O_2_ in the deeper cortical and outer medullary tissue in DAPA treated diabetic rats. No significant differences were observed between the MAPs, between groups (Figure 5, upper panel).

### 3.4 SGLT2 inhibition modulates hypoxia‐related gene expression

**FIGURE 6:**
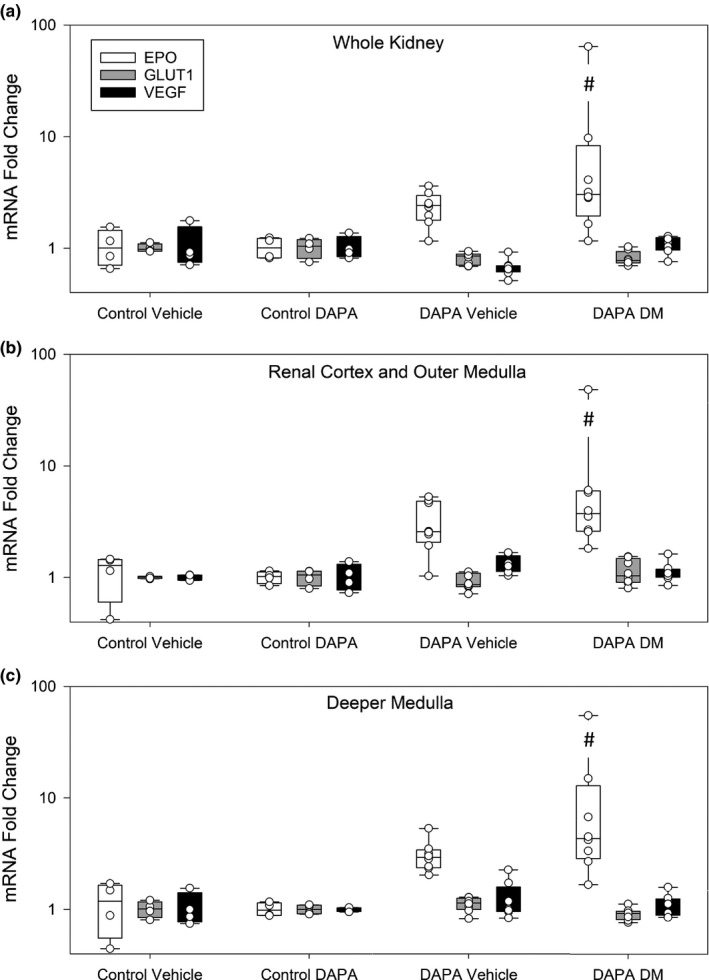
Quantitative polymerase chain reaction of hypoxia inducible factor responsive genes. Erythropoietin (EPO), vascular endothelial growth factor (VEGF) and glucose transporter 1 (GLUT 1) mRNA levels were assessed in three independent tissue samples from different regions of the kidney. EPO RNA, but not GLUT 1 or VEGF, RNA levels tended to be higher in the diabetic kidney. EPO RNA levels were highest in diabetic rats treated with dapagliflozin (DAPA) for all three tissue samples (#; *p* < 0.041 vs. Control Vehicle and Control DAPA; one‐way ANOVA on Ranks). There were no significant differences between GLUT1 and VEGF expression

To ascertain the cellular response to the changes in kidney tissue oxygen tension we focused on three hypoxia sensitive genes, the transcription of which are responsive to changes in pO_2_ via hypoxia inducible factor (HIF): EPO, VEGF and GLUT 1. EPO, one of the most hypoxia sensitive genes (Neuen et al., 2019), is expressed by pericapillary interstitial fibroblast‐like cells at the cortico‐medullary junction (Johannes et al., 2007). EPO mRNA levels were increased in three separate regional samples of the kidneys of diabetic rats treated with DAPA compared with non‐diabetic rats (*p* < 0.041; Figure 6). No changes in other hypoxia‐inducible factor induced molecules, including VEGF and GLUT1 were detected.

### 3.5 SGLT2 inhibition results in changes to RBC parameters

**FIGURE 7:**
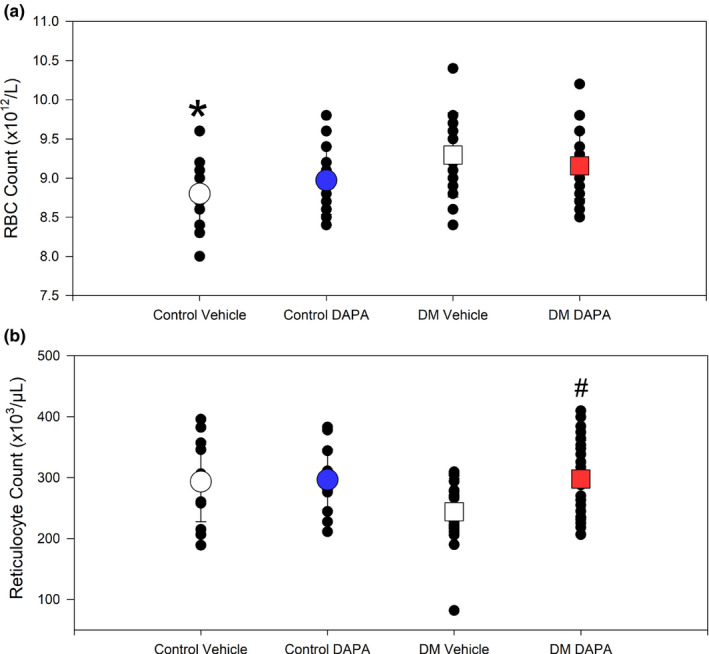
Effect of dapagliflozin (DAPA) treatment on red blood cell and reticulocyte counts in non‐diabetic and diabetic rats. (a) Non‐diabetic rats treated with vehicle had significantly lower red blood cell (RBC) count relative to diabetes mellitus (DM) vehicle (*, *p* < 0.018; one‐way ANOVA). (b) An increase in reticulocyte counts was observed in DAPA treated diabetic rats, relative to vehicle‐treated diabetic rats (#, *p* = 0.024; one‐way ANOVA; *n* = 14–24)

Total RBC counts were not different between DAPA and vehicle‐treated diabetic rats, but were significantly higher than control vehicle values (*p* < 0.018; Figure 7, upper panel). Diabetic rats treated with DAPA had significantly increased reticulocyte counts relative to diabetic rats treated with vehicle (Figure 7; *p*<0.025).

## 4 DISCUSSION

The mechanisms underlying the salutary effects of SGLT2 inhibition remain elusive. While reduction in renal oxygen consumption and relief from hypoxia has been proposed to contribute, *in vivo* studies employing a selective SGLT2 inhibitor have been lacking (Hesp et al., 2020). By conducting a study with a widely‐used SGLT2 inhibitor in a well‐established experimental model of diabetes mellitus, we sought to fill this knowledge gap. Rather than confirming SGLT2 inhibition improved tissue oxygen tension, DAPA was shown to reduce microvascular kidney oxygen tension in an area that spans the cortex and outermost medulla, in diabetic rats. These findings likely reflect the interplay of three SGLT2‐mediated effects: (1) reduced post‐glomerular perfusion secondary to afferent arteriolar constriction induced by TGF; (2) decreased sodium reabsorption in the early proximal tubule; and (3) increased sodium reabsorption more distally including the S3 segment of the proximal tubule. The lack of any effect of SGLT2 inhibitors on tissue oxygen tension in the superficial renal cortex that includes little if any S3 segments is consistent with a balanced effect on oxygen consumption and delivery within this region.

Collectively, our data demonstrate that treatment of diabetic rats with an SGLT2 inhibitor, reduced GFR, decreased deeper renal cortical, and outer medullary microvascular P_k_O_2_, increased expression of the HIF‐dependent molecule EPO and increased systemic RBC reticulocyte count. These changes were not observed in SGLT2 treated non‐diabetic rats. Furthermore, the lack of a change in superficial microvascular renal cortical P_k_O_2_ serves as an internal negative control, demonstrating the lack of effect of SGLT2 inhibition in this region of the kidney. The finding of a decrease in P_k_O_2_ in adjacent deeper cortical and outer medullary tissue suggests DAPA treatment affected this region differentially; possibly due to its relatively higher metabolic activity. The overall balance between oxygen delivery and consumption resulted in a region‐specific decrease in P_k_O_2_. The increase in EPO mRNA level and reticulocyte count observed in DAPA treated diabetic rats represents an appropriate compensatory response to the measured decrease in renal P_k_O_2_. This experimental finding is consistent with the findings of increased systemic EPO levels and hemoglobin concentration (Hb)/hematocrit, in type 2 diabetes mellitus patients treated with this class of medication (Mazer et al., 2020; Verma et al., 2019).

These results provide insights into the overwhelming clinical data demonstrating that SGLT‐2 inhibitors are renal protective (Neuen et al., 2019; Perkovic et al., 2019). The small decrease in renal microvascular P_k_O_2_ may represent a condition of “physiologic hypoxia” which we speculate may activate an AMP kinase dependent mechanism to protect renal cells via inhibition of cellular senescence (van Vliet et al., 2021). Thus, with dapagliflozin treatment of diabetic rats, the cells of the deeper renal cortex and outer medulla, may be protected by such “physiologic hypoxia”. This mechanism has been proposed to explain the renal protective effect of dapagliflozin in a model of ischemia reperfusion injury (Chang et al., 2016). In addition, our finding of increased EPO mRNA levels in the kidney of SGLT‐2 inhibitor treated diabetic rats, is consistent with a reduction in P_k_O_2_ and increased HIF expression (not measured). HIF dependent gene expression have been proposed to initiate renal protective mechanisms in the kidney (Chang et al., 2016; van Vliet et al., 2021). These findings are consistent with clinical studies identifying that systemic EPO levels are increased in patients receiving SGLT‐2 inhibitors (Mazer et al., 2020; Verma et al., 2019). It has been estimated that the associated increase in systemic hematocrit (~5%) may be responsible for as much as 50% of the increase in patient survival observed in clinical trials (Inzucchi et al., 2018). Thus, mild renal hypoxia may protect the kidney, while initiating a systemic response (EPO derived erythrogenesis), which may improve systemic oxygen delivery and organism survival (Inzucchi et al., 2018; Li et al., 2020).

With respect to other studies which report measurement of renal P_k_O_2_ utilizing different experimental models; these studies have a number of limitations. One study reports PO values utilizing a method that measure very low cellular oxygen levels (<10 mmHg, pimidazole; Kamezaki et al., 2018) such low tissue PO_2_ values do not occur under physiological conditions in the region of cortical medullary junction (Abrahamson et al., 2020; Ast & Mootha, 2019; Johannes et al., 2007; O'Neill et al., 2015). Another study assessed the effect of SGLT‐2 inhibitors in vitro, in cellular models that do not reflect the complex physiological dynamic of oxygen supply and demand within the kidney (Bessho et al., 2019). Finally, another study measured the effect of acute administration of an SGLT1 and 2 inhibitor (O'Neill et al., 2015), which has little relevance on the impact of long term therapy with specific SGLT‐2 inhibition in animals or patients with established diabetes.

Kamezaki et al. (2018), utilized a pimonidazole compound which measure changes in intracellular PO_2_ below 10 mmHg (Gross et al., 1995; Ragnum et al., 2015). The pimonidazole method predominantly measures kidney regions in the deep medulla, which have very low PO_2_ values. These measured values are well below the physiologically relevant levels of PO_2_ at the cortical medullary junction which are consistently measured at P_k_O_2_ values near 40 mmHg (Abrahamson et al., 2020; Johannes et al., 2007; O'Neill et al., 2015). Published data demonstrate that pimonidazole staining (PO_2_ < 10 mmHg) remained unchanged in the renal medulla after ipragliflozin administration (1–2 weeks; Kamezaki et al., 2018). By contrast, reduced levels of staining were observed in the renal cortex of diabetic mice after ipragliflozin treatment. Measurement of an increase in PO_2_ utilizing this method should not be possible in this region of relatively high microvascular and tissue PO_2_, and call into question the validity of this finding. Other cited studies have measured superficial renal cortical tissue PO_2_ values near 40 mmHg and renal medullary values near 25–30 mmHg (Clarke electrode) in a STZ rat model of diabetes (O'Neill et al., 2015). These vital renal PO_2_ values, agree with our measurements, and suggest that the physiological renal tissue PO_2_ values are much higher than 10 mmHg. Data from O'Neil et al., differ from the current study in that they assessed the impact of acute SGLT 1 and 2 inhibition (phlorizin); not specific SGLT‐2 inhibition, in a similar model of diabetes. Their data demonstrate an increase in renal cortical tissue oxygen PO_2_ after acute SGLT‐1,2 inhibition (~40 to 50 mmHg) and a small decrease in renal medullary tissue PO_2_ (~30 to 25 mmHg). These measurements do not refute our current measure of vital microvascular PO_2_ which demonstrate a reduction in deeper renal cortical and outer medullary microvascular PO_2_ in DAPA treated diabetic rats, as prediced by Hesp et al. (2020). By contrast, measurements in the current study do not demonstrate any change in superficial renal cortical PO_2_, suggesting that only the deeper region of the kidney are influenced by SGLT‐2 inhibition in diabetic rats.

The proximal tubule consists of three contiguous segments which can be distinguished by their ultrastructural appearance, their transporter function and their location within the kidney. The first two segments, S1 and S2 that express SGLT2, constitute the pars convoluta that lie entirely within the cortex. The third segment, S3 or pars recta that expresses SGLT1 but not SGLT2, lies in the deep inner cortex and outer medulla (Fenton et al., 2016). The reduction in P_k_O_2_ in the region that encompasses the entire cortex and OSOM (red light) but not in the superficial cortex (blue light) suggests that a shift of sodium reabsorption to the S3 segment with SGLT2 inhibition increases its oxygen consumption. Such findings would be consistent with the increased glucose transport that occurs in the S3 with SGLT2 inhibition where the proportion of filtered glucose reabsorbed by this segment increases from 3% to over 40% (Hesp et al., 2020).

Approximately 80% of the kidney's oxygen consumption is devoted to sodium reabsorption, so changes in its filtration and reabsorption will be expected to alter oxygen consumption (Layton et al., 2016). While sodium reabsorption in the thick ascending loop is more energy efficient (12 molecules of sodium per ATP) than the proximal tubule (nine molecules of sodium per ATP), the distal tubule, the primary site of sodium regulation, is far less energy‐efficient, transporting only three molecules of sodium per molecule of ATP (Mount, 2016). Accordingly, even minor changes in sodium delivery to the distal nephron can have a substantial impact on oxygen consumption, consistent with the finding that the hypoxia sensitive EPO was up regulated in dapagliflozin treated diabetic animals.

To the best of our knowledge, the current study is the first to examine kidney oxygenation with a selective SGTL2 inhibitor. Previous investigations of the effects of SGLT inhibition on kidney oxygenation have been reported, but importantly, these relied on the dual SGLT1/2 inhibitor, phlorizin (Körner et al., 1994; O'Neill et al., 2015). However, given the differential distribution of SGLT1 and ‐2 within the kidney, the effects of dual inhibition cannot be extrapolated to selective SGLT2 inhibition, as shown by our current findings. While modeling studies add an important dimension to our understanding, they may not take into account the effects on postglomerular perfusion that develop as a consequence of TGF‐induced afferent arteriolar constriction (Layton et al., 2016). Here, by directly measuring P_k_O_2_, we provide a summative assessment of these opposing SGLT2‐mediated effects, demonstrating reduced microvascular pO_2_ in the deeper cortex and outermost medulla with a neutral effect in the superficial cortex.

In contrast to its effects in the diabetic setting, SGLT blockade has been shown to have substantially less impact in non‐diabetic rats with regard to effects on both ion transport and TGF activity (Thomson et al., 2012; Vallon et al., 1999). This is consistent with our experimental findings in that SGLT2 inhibition had little effect on non‐diabetic animals. Corroborating our findings in rats, a recent report of the effects using the SGLT2 inhibitor, empagliflozin, in humans, did not find any effect on kidney oxygenation, as assessed by blood oxygenationlevel‐dependent magnetic resonance imaging (Zanchi et al., 2020). The absence of an effect on kidney oxygenation in non‐diabetic rodents or humans, despite a robust response on CKD progression in non‐diabetic patients in a recent trial, argues against the nephroprotective effects of SGLT2 inhibition being mediated by improved kidney oxygenation (Heerspink et al., 2020).

The strengths of the current study center on the use of robust technology to precisely determine tissue oxygenation. While our study does not provide clear mechanistic evidence, it does provide accurate data about the renal microvascular P_k_O_2_ measures in this model of SGLT‐2 treatment in a relevant model of diabetes. In our experimental model, SGLT‐2 treatment, corrects the diabetes induced increase in GFR, and reduces renal cortical and outer medullary PO_2_. In using surface light and not needing to puncture the kidney, as is undertaken using the Clark electrode, the potential confounding effects of atmospheric contamination and tissue damage are eliminated.

Our current study does, however, have several limitations. First and foremost, it is a study on animals and not humans and one in which diabetes was induced experimentally. In addition, the level of hyperglycemia in the experimental setting was much greater than would usually be encountered clinically. As a consequence of hyperglycemia, vehicle‐treated diabetic rats had marked glucosuria and polyuria; and while glycemia was substantially improved by dapagliflozin, SGLT2 inhibition led to glucosuria and polyuria that were similar to those receiving vehicle only. Our measurements provide similar levels of microvascular PO_2_ for vehicle‐treated control and diabetic rats, which may reflect that these measures are at an early point of the gradient for tissue oxygen delivery. The finding of elevated EPO mRNA levels in diabetic rats, is consistent with the experimental observation of renal hypoxia in deeper medullary (but not cortical) tissue in diabetic rats measured by oxygen electrodes (O'Neil et al. 2015).^2007^

## 5 CONCLUSIONS

Our current findings indicate that improvement in kidney oxygenation is unlikely to underlie the nephroprotective effects of SGLT2 inhibition. We have observed that SGLT2 inhibition reduced GFR and microvascular tissue PO_2_ in a region of the kidney which is hypoxia responsive. These data are combined with evidence of increased renal EPO mRNA levels (renal tissue hypoxia) and an increase in reticulocyte, suggesting that a decrease in renal PO_2_ may explain the observed erythrogenesis in clinical studies in patients treated with this drug class (Januzzi et al., 2017; Mazer et al., 2020; McMurray et al., 2019; Verma et al., 2019; Zinman et al., 2015).

## DISCLOSURE

Funding for this study was contributed, in part, by an investigator‐initiated grant from Astra Zeneca that had no part in the design, conduct, writing or interpretation of the study. REG reports receiving other research grants to his institution from AstraZeneca and Boehringer Ingelheim; serving on advisory panels for AstraZeneca, Boehringer Ingelheim, and Janssen; receiving CME speaker honoraria from AstraZeneca, Bayer, Boehringer Ingelheim, and Janssen, all unrelated to the current study. He also reports being a shareholder in Certa Therapeutics, OccuRx and Fibrocor Therapeutics and is CSO of Fibrocor Therapeutics. KAC reports receiving research grants to his institution from AstraZeneca and Boehringer Ingelheim; serving on advisory panels for AstraZeneca, Boehringer Ingelheim, and Janssen; receiving CME speaker honoraria from AstraZeneca, Bayer, Boehringer Ingelheim, and Janssen, all unrelated to the current study. RGE reports receiving consulting fees from Medtronic Inc, unrelated to the current study.

## AUTHOR CONTRIBUTION

Gregory M.T. Hare, Yanling Zhang, Kyle Chin, David F. Wilson, Sergei A. Vinogradov, Kim A. Connelly, C. David Mazer, Roger G. Evans and Richard E. Gilbert conceived and designed research; Gregory M.T. Hare, Yanling Zhang, Kyle Chin, Kerri Thai, Evelyn Jacobs, Melina P. Cazorla‐Bak and Linda Nghiem performed experiments; Gregory M.T. Hare, Yanling Zhang, Kyle Chin, Kerri Thai, Evelyn Jacobs, Melina P. Cazorla‐Bak, Linda Nghiem, Kim A. Connelly, C. David Mazer, Roger G. Evans and Richard E. Gilbert analyzed data; all authors interpreted results of experiments; Gregory M.T. Hare, Yanling Zhang, Kyle Chin, Kerri Thai, E.J., Melina P. Cazorla‐Bak, Linda Nghiem and R.E.G. prepared figures; Gregory M.T. Hare, Yanling Zhang, Kyle Chin, Kim A. Connelly, C. David Mazer, Roger G. Evans and Richard E. Gilbert drafted manuscript; all authors edited, revised and approved final version of manuscript.

## Data Availability

The data are available from the corresponding author on reasonable request.
